# Nutritional Management and Outcomes in Malnourished Medical Inpatients in 2020: The Evidence Is Growing!

**DOI:** 10.3390/jcm9010027

**Published:** 2019-12-20

**Authors:** Philipp Schuetz, Zeno Stanga

**Affiliations:** 1 Department of Endocrinology, Diabetes and Clinical Nutrition, University Department of Internal Medicine, Kantonsspital, CH-5001 Aarau, Switzerland; 2 Medical Faculty of the University of Basel, CH-4056 Basel, Switzerland; 3 Department of Diabetes, Endocrinology, Nutritional Medicine and Metabolism, Inselspital, Bern University Hospital, and University of Bern, CH-3008 Bern, Switzerland; Zeno.Stanga@insel.ch

Access to adequate food is a fundamental human right. There is no doubt that nutrition is essential in maintaining health and preventing or treating disease. Particularly when patients are affected by disease-related malnutrition, their risk of adverse clinical outcomes increases significantly and optimizing nutritional support becomes mandatory [1,2].

There is ongoing debate about what constitutes an optimal nutritional care process in terms of screening, assessment, and use of nutritional support in different patient populations. Issues include dose and quality of proteins and total energy, route of delivery, and whether or how nutritional support needs to be adjusted for specific medical and metabolic conditions. As we move toward personalized medicine, which is based on patients’ individual needs, an understanding of these different factors is important. Well-planned clinical studies of high methodological quality are needed to develop the best approach to providing individualized nutritional support [3].

Historically, much of the evidence regarding effects of nutritional support has come from small interventional trials and observational studies with cross-sectional or cohort-study designs, whereas there was an important lack of large-scale randomized interventional research, which is needed to establish causal effects rather than just statistical associations [4,5]. As a consequence, the medical community has struggled to design efficient, evidence-based approaches for the prevention and treatment of malnutrition [4].

Recently, however, the results of several high-quality trials have provided important new insights that advance nutritional science significantly and translate nutrition research into practice [6]. Regarding prevention of cardiovascular disease through nutrition, the PREDIMED (Prevención con Dieta Mediterránea) trial provided strong evidence that a Mediterranean diet supplemented with extra-virgin olive oil or with mixed nuts reduces the risk of cardiovascular and metabolic disease by about 30% over five years [7]. Regarding the use of clinical nutrition in patients at nutritional risk or with established malnutrition, two trials found nutritional support to be highly effective. First, the multicenter, randomized, placebo-controlled NOURISH trial (Nutrition effect On Unplanned ReadmIssions and Survival in Hospitalized patients) including 652 older adults affected by malnutrition found that a high-protein oral nutritional supplement containing beta-hydroxy-beta-methylbutyrate was associated with a significant reduction in 90 day mortality, with a number needed to treat (NNT) of 20 [8]. Second, the EFFORT (Effect of early nutritional support on Frailty, Functional Outcomes and Recovery of malnourished medical inpatients Trial) including 2028 medical inpatients at nutritional risk in eight Swiss hospitals showed that protocol-guided individualized nutritional support designed to achieve protein and energy targets results in significantly lower rates of severe complications (NNT = 25) and mortality (NNT = 37) compared to regular hospital food [9,10]. Moreover, functional decline was significantly lower, and quality of life as well as activities of daily living significantly improved. A recent meta-analysis including these two trials and several other trials also came to the conclusion that nutritional support in the malnourished medical inpatient population reduces the risk for both, mortality and hospital readmission by about 25% [6].

Evidence-based medicine is an approach used to optimize decision-making by emphasizing evidence from properly designed and well-conducted research, typically randomized trials and meta-analyses from such trials. With the growing number of high-quality trials such as the ones mentioned above, we are increasingly able to practice “evidence-based clinical nutrition” and to adapt nutrition to the individual patient’s needs. This Special Issue of the *Journal of Clinical Medicine* (JCM) focuses on a topic that is critical for hospitals today: “Nutritional Management and Outcomes in Malnourished Medical Inpatients”. This special edition presents a number of reviews and original research articles in the field of nutritional management and clinical outcomes in malnourished medical inpatients. Twenty-six important articles illustrate the different facets of this complex and timely topic.

The articles included cover the process of nutritional care, including screening tools to identify nutritional risk (Nutritional risk screening and assessment [11]), patient muscle mass assessment including bioimpedance analysis (Clinical value of muscle mass assessment in clinical conditions associated with malnutrition [12]; Decreased bioelectrical impedance phase angle in hospitalized children and adolescents with newly diagnosed type 1 diabetes: a case-control study [13]), nutritional biomarkers (Nutritional laboratory markers in malnutrition [14]), nutritional therapy planning (Indirect calorimetry in clinical practice [15]; Micronutrient deficiencies in medical and surgical inpatients [16]), use of nutritional support overall (Efficacy and efficiency of nutritional support teams [17]; Challenges and perspectives in nutritional counselling and nursing: a narrative review [18]) and in specific patient populations (e.g., medical patients, critical care patients, geriatric patients, oncologic patients, patients after allogenic stem cell transplantation, patients with dysphagia or eating disorders, as well as the nutritional challenges associated with metabolic disorders) (Nutritional management of medical inpatients [19]; Medical nutrition therapy in critically ill patients treated on intensive and intermediate care units: a literature review [20]; Metabolic and nutritional characteristics of long-stay critically ill patients [21]; Protein intake, nutritional status, and outcomes in intensive care unit survivors: a single-center cohort study [22]; Early supplemental parenteral nutrition in critically ill children: an update [23]; Management of malnutrition in older patients—current approaches, evidence, and open questions [24]; Nutrition in cancer patients [25]; Management of dehydration in patients suffering swallowing difficulties [26]; Nutrition in gastrointestinal diseases: liver, pancreatic, and inflammatory bowel diseases [27]; Nutritional management and outcomes in malnourished medical inpatients: anorexia nervosa [28]; Nutritional challenges in metabolic syndrome [29]; Nutritional challenges in patients with advanced liver cirrhosis [30]). Potential complications of nutritional interventions, such as refeeding syndrome (Management of refeeding syndrome in medical inpatients [31]), and treatment challenges posed by gastric motility disorders are discussed (Gastroparesis and dumping syndrome: current concepts and management [32]). Economic aspects of nutritional management (Economic challenges in nutritional management [33]) and specific considerations such as pharmaceutical/therapeutic aspects of artificial nutrition are additionally reviewed (Management of glucose control in non-critically ill, hospitalized patients receiving parenteral and/or enteral nutrition: a systematic review [34]; Pharmaceutical aspects of artificial nutrition [35]). 

Last but not least, the call for political commitment in the treatment of malnutrition is of key importance. Health care institutions and associations must be mobilized to take action against malnutrition by expanding information and public-awareness-raising campaigns, adopting supportive policies, as well as allocating resources (Hospital malnutrition, a call for political action: a public health and nutritionDay perspective [36]). Stakeholders including political organizations, nutritional social networks, and researchers will be valuable promoters of this important political priority in the future.

Understanding the optimal use of nutritional therapy is highly complex. Timing, route of delivery, and the amount and type of nutrients all play important roles and potentially affect patient outcomes. Recent trials provide important information to strengthen the evidence regarding the use of nutritional therapy in specific patient populations, but there are still important questions to be addressed by robust clinical trials in the future. It is now important to incorporate these recent findings into clinical practice in order to ensure that our patients receive high-quality, safe, and optimal care.
